# Eczema Herpeticum in children with burns

**DOI:** 10.12669/pjms.37.2.3642

**Published:** 2021

**Authors:** Fatima Naumeri, Sushil Rijal, Abdul Rehman Rashid, Hafiz Mahmood Ahmad

**Affiliations:** 1 Dr. Fatima Naumeri, MCPS, FCPS. Department of Pediatric Surgery, King Edward Medical University/Mayo Hospital, Lahore, Pakistan; 2 Dr. Sushil Rijal, MBBS. Department of Pediatric Surgery, King Edward Medical University/Mayo Hospital, Lahore, Pakistan; 3 Dr. Abdul Rehman Rashid, MBBS Department of Pediatric Surgery, King Edward Medical University/Mayo Hospital, Lahore, Pakistan; 4 Dr. Hafiz Mahmood Ahmad, FCPS Department of Pediatric Surgery, King Edward Medical University/Mayo Hospital, Lahore, Pakistan

**Keywords:** burn, children, eczema herpeticum, treatment, incidence, outcome

## Abstract

**Background & Objective:**

Eczema herpeticum (EH) is a disseminated viral infection occurring in pre-existing skin conditions and burns. The objective of this study was to determine the frequency, treatment, and outcome of EH in pediatric burn patients.

**Methods:**

This retrospective study was conducted in the pediatric surgery department, King Edward Medical University/ Mayo Hospital, Lahore, from October 2015 to July 2018 after ethical approval. All pediatric burn patients diagnosed with EH and not sensitive to Acyclovir or suffering from chemical burns were enrolled in the study. Diagnosis was confirmed by presence of umbilicated lesions in burnt area and a positive Tzanck smear. Intravenous acyclovir and supportive treatment was started. Mortality, development of contractures, length of hospital stay/ time for wound healing, re-activation of EH was calculated.

**Results:**

Out of 3958 admitted pediatric burn patients, 94(2.4%) developed EH. Girls were 58(61.7%) and boys were 36(38.3%). Mean age was 5.16 ±2.88 years. Scald burn was in 43(45.7%) patients, flame burn in 48(51.1%) patients, and electric flash burn in 3(3.2%) patients. Mean TBSA was 21.74+10.38%. Vesicular eruptions settled in 92 (97.9%) patients after treatment with acyclovir. Mean duration of treatment was 19.89+ 8.9 days and hospital stay was 29.84+ 16.98 days. Twenty three patients (24.5%) developed contractures and two patients (2.1%) developed disseminated EH and expired. Six (6.4%) patients had re-activation of EH.

**Conclusion:**

EH occurred in 2.4% of admitted pediatric burn patients. Intravenous acyclovir was successful in 97.9% of the patients, although 2.1% developed disseminated EH and expired. Re-activation occurred in 6.4% of the cases and was associated with prolonged hospital stay.

## INTRODUCTION

Eczema Herpeticum, or Kaposi-Juliusberg varicelliform eruption, is a disseminated viral infection characterized by “painful, pruritic umbilicated vesiculopustular eruptions”. It commonly occurs in young patients with pre existing skin conditions like atopic dermatitis, burns, and eczema. Patients who are immune-compromised are at a higher risk. This rare disease has widespread morbidity and mortality, associated with bacterial sepsis, viremia and involvement of multiple organs. The exact incidence of disease is not known. In USA annual incidence of hospitalization in children due to Eczema Herpeticum (EH) ranges from 4.03 to 7.30 per million.^1^-^3^

Eczema Herpeticum (EH) is mainly caused by Herpes Simplex Virus (HSV 1) type 1, although Herpes Simplex Virus (HSV) types 2, varicella zoster virus (VZV), cytomegalovirus (CMV) are found in some cases. Patients suffering from EH develop characteristic umbilicated skin eruptions and systemic symptoms like fever, malaise and lymphadenitis. Activation of viral infection like HSV, CMV, and VZV play decisive role in morbidity and mortality in burn patients.^1^,^4^

The diagnosis of EH is mainly based on clinical examination of skin eruptions and laboratory tests like Tzanck smear, viral culture, direct immunofluorescence, skin biopsy and polymerase chain reaction (PCR). Once the disease is diagnosed, anti-viral drugs are given orally or intravenously with topical application to treat EH.^5^

Limited data is available in literature regarding the incidence and outcome of EH in pediatric burn patient. However, one study done in United States showed 0.1% mortality among children admitted in hospital.^2^ Whereas mortality rate is up to 2.7% in severely burned children with Herpes virus infection.^3^

One study in adult burn patients reported EH frequency of 4.2% with mortality rate 11%.^6^Although, HSV infection is not straightforwardly associated to higher mortality but it impairs wound healing and may lead to sepsis.^4^ This study was designed to determine the frequency, treatment and outcome of EH in pediatric burn patients.

## METHODS

**Fig.1:**
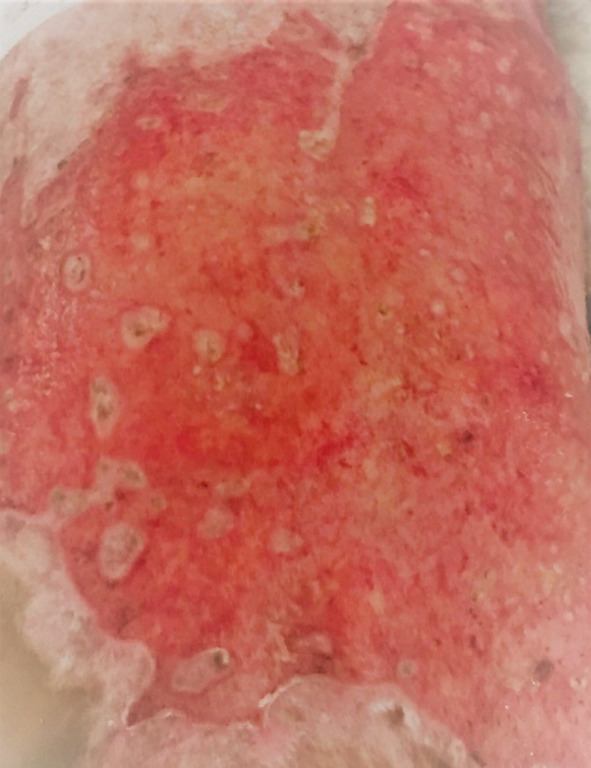
Presence of Umbilicated Lesion of Eczema Herpeticum in Burnt Wound.

This retrospective study was conducted at the department of Pediatric Surgery, King Edward Medical University/ Mayo Hospital, Lahore, from October 2015 to July 2018. Ethical approval was taken from Institutional Review board (No. 532/RC/KEMU dated 29/07/2020). All burn patients with age less than 13 years and diagnosed with EH were included, using census sampling technique. Patients who were sensitive to Acyclovir or presented after chemical burns were excluded. Diagnosis of EH was confirmed by a dermatologist on the basis of presence of umbilicated lesions in previously burnt area (Fig.1) and a positive Tzanck smear. For Tzanck smear, scrapping of suspected lesion was done and stained with Giemsa stain. If the smear revealed multinucleated keratinocytes/ giant cells then it was labeled as ‘positive’.

**Fig.2:**
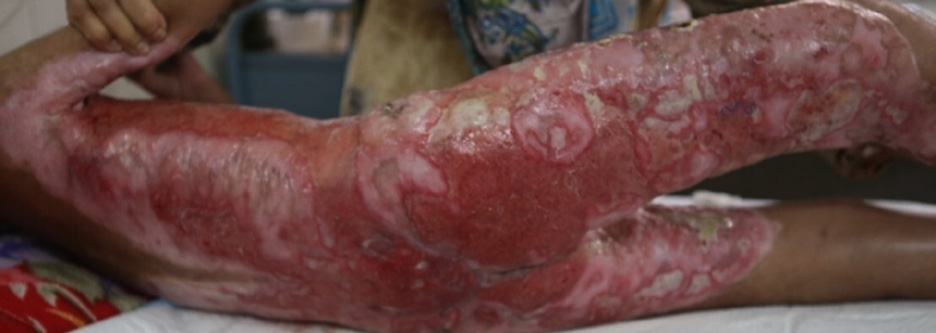
Partially Healed Lesion of Eczema Herpeticum with super added Bacterial infection seen as purulent discharge.

Management was started immediately after clinical diagnosis, and intravenous fluids, painkillers, Proton Pump inhibitors and empirical broad spectrum antibiotics (based on ward antibiogram^7^) were given initially. Later on culture specific antibiotics were started. Partial healing of lesions and purulent discharge indicating superadded bacterial infection and need of culture specific antibiotics as shown in Fig.2.

Antiviral injection acyclovir 10mg/kg/dose thrice a day was given and continued till complete healing of skin lesions and a negative Tzanck smear. Topically acyclovir and silver sulfadiazine was applied. Tzanck smear was repeatedly every fortnightly to rule out re-activation of EH, once the lesions were settled. Debridement was planned only after negative Tzanck smear and skin grafting was done on subsequent days once wound culture was negative, and wound was healthy. Hemoglobin levels were maintained over 8gm/dl during the hospital stay and blood was transfused when required. If patient developed severe allergic reactions to Acyclovir, developed diarrhea or derangement of liver functions, Famciclovir was started.

Variables reviewed were patient characteristics (age, gender, weight, hemoglobin, type of burn, total body surface area [TBSA] involved), co-morbidities such as previous skin disease, features of EH (fever, skin eruptions, malaise, lymphadenitis), treatment protocols (duration of acyclovir, healing of eruptions, need of blood transfusions) and outcome (wound healing time, length of hospital stay, contractures, re-activation of EH and/or mortality).

Data analysis was done by using SPSS 23. Qualitative data (gender, type of burn, TBSA, previous skin disease, fever, malaise, and lymphadenitis, need of transfusion, contractures, re-activation of EH and mortality) was analyzed as frequency and percentage. Quantitative data (age, weight, hemoglobin, duration of acyclovir, wound healing time, length of hospital stay) was analyzed as mean and standard deviation. Logistic regression was used to analyze association of re-activation of EH and length of hospital stay, mechanism of burn, TBSA, gender, and age; p-value of <0.05 was taken as significant.

## RESULTS

Of the 7556 burn patients presenting in pediatric surgical emergency during the study period, 3958 patients were admitted. Ninety four (2.4%) patients developed eczema herpeticum (EH) in the admitted burn patients. Of them 58(61.7%) were female and 36(38.3%) were male. The mean age of patients was 5.16 ±2.88 years (range 0.33 to12 years). Mean weight of patients was 15.88±5.78 kilogram (range 7 to 35 kg).The mean hemoglobin was 9.26±1.86 g/dl (range 5.1 to 15.8 g/dl).

Thirty two (34%) presented within a day of burn and 62(66%) presented late. Type of burn was scald burn in 43(45.7%) patients, flame burn in 48(51.1%) patients, and electric flash burn in 3(3.2%) patients.

**Fig.3:**
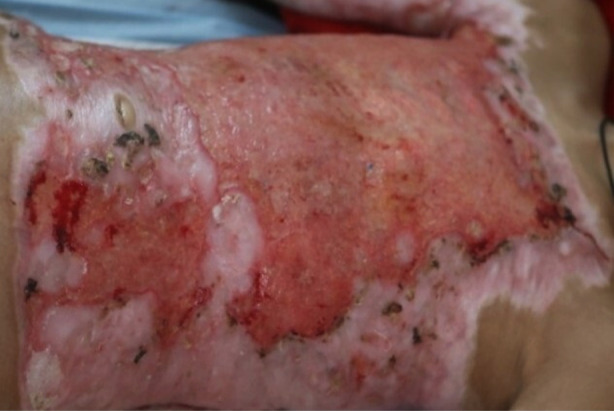
Healed Umbilicated lesions of Eczema Herpeticum.

Mean TBSA was 21.74+10.38%. (Minimum was 5% and Maximum was 60%). All (100%) of the patients had fever during their hospital stay. Vesicular eruptions settled in 92 (97.9%) patients after successful treatment with acyclovir. Fig.3 shows healed EH lesions after antiviral therapy.

Mean duration of treatment was 19.89+8.9 days. (Minimum 7 days to Maximum 48 days). Blood transfusion was done in 87(92.6%) patients and 7(7.4%) did not require any transfusion during hospital stay. None of the patients gave past history of any skin disease or developed lymphadenitis. No patient developed reaction to Acyclovir.

Mean length of hospital stay was 29.84+16.98 days. Out of 94 patients, 23 patients (24.5%) developed contractures and two patients (2.1%) developed disseminated EH and expired. Six (6.4%) patients had re-activation of EH. There was no association of re-activation of EH and age (p value 0.07), gender (p value 0.3), TBSA (p value 0.1). Re-activation was associated with longer than one month of hospital stay (p value=0.0001).

## DISCUSSION

The frequency of EH in admitted burn patients in this study was 2.4%. Other studies have shown incidence of EH is 4.5% in pediatric population 3 and 4.2% in adult population^8^. EH can occur in individuals of all ages but is seen more commonly in pediatric patients, especially in children two to three years of age^8^ but in our study mean age is 5.16 years.

Activation of viral infection after burns can occur and lead to eczema herpeticum (EH) in admitted patients. Due to reactivation of HSV, gamma interferon has been prescribed in cases of EH. Though a study conducted by Darji K et al concluded that gamma interferon have no role in improving EH or preventing its’ relapse^9^.

Till now no studies have demonstrated the evaluation of Acyclovir therapy in pediatric burn patients with EH. Although, its use has been described in children hospitalized with EH.^3^ In most cases, acyclovir should be administered intravenously (1,500 mg/m2 per day in three divided doses or 15 to 30 mg/kg per day in three divided doses for seven days) though children who appear well and have limited disease can be treated orally (1,000 to 1,200 mg/d in three to five divided doses for five to seven day). For more severely affected children, acyclovir may be given up to 10 days or at least until no new lesions emerge^8^. In this study, intravenous acyclovir was given till lesions settled. Duration of hospital stay was 29.84+16.98 days and duration of acyclovir treatment was 19.89+8.9 days. This duration is longer than mean duration of treatment (9.8±3.1 days) mentioned by Luca et al.^10^

In cases of acyclovir resistance, Foscarnet is used.^11^,^12^ Valacyclovir and Famciclovir are recommended in patients not responding to acyclovir due to their better oral absorption.^11^-^13^ However Valacyclovir is not recommended in less than 12 years of age.^11^ Our dermatologists advise Famciclovir for any patient who develops allergic reaction to acyclovir, though no patient in this study needed it. Re-activation occurred in 6.4% cases in this study and needed acyclovir for a further seven days. No association of re-activation could be noted with age, gender, mechanism of burn or TBSA. Re-activation was associated with prolonged hospital stay (p value 0.0001). A study of factors associated with recurrence of EH revealed recurrence rate of 8.9% within first month and rate of repeat episode as 16% after first month, with increased association in hospitalized patients and no association with age, gender, type of treatment or length of hospital stay.^10^ These findings are similar to our study except length of hospital stay.

Additional measures of EH treatment include local skin care, administration of antipyretic and analgesic medications, provision of other general supportive care, such as intravenous hydration, electrolytes, and blood products. In this study topical acyclovir and silver sulfadiazine was applied. In healed areas topical corticosteroids may be used. Use of corticosteroids topically doesn’t affect outcome of EH, while systemic steroids may suppress immunity and lead to dissemination of EH.^14^,^15^ In this study, generally Hemoglobin levels were maintained at 8 g/dl and 92.6% patients needed blood transfusion.

Appropriate antibiotics should be administered when secondary bacterial infection is suspected.^14^,^16^ Luca et al^10^ administered antibiotics in 77.5% patients who developed secondary infection. In this study all patients needed antibiotic coverage. In a study conducted by Aronson PL et al^17^ administering empirical antibiotics doesn’t affect length of hospital stay except in cases of bacteremia, though earlier acyclovir administration results in earlier discharge^16^. A local study showed that Staphylococcus Aureus is the main pathogen infecting burn patients.^18^ Our ward antibiogram also suggest similar findings, though this study is limited in regards to observing growth patterns in burn patients.^7^

Due to easy availability we preferred using Tzanck smear. More specific investigations include serum herpes simplex virus IgM/IgG levels, transmission electron microscopy for identification of viral particles, PCR for viral DNA, viral culture, and direct fluorescence antibody staining and in atypical cases skin biopsy is required.^13^,^19^ In burns, EH may be difficult to differentiate from shingles though lack of involvement of a particular dermatome is helpful in differentiating the two conditions clinically and PCR can provide the definitive diagnosis.^20^

We also isolated the burn patients diagnosed with EH in separate room with separate bath area. Health worker dealing with infected patients were given separate linen, gloves, dressing trolleys/material and hand washing area. For those, who need debridement, sterilized surgical instruments were used in patients and Operation Theater was fumigated after use. Isolated room, beds, chairs, tables, floor and medical instruments used like sphygmomanometer, thermometer, IV stands were routinely decontaminated to prevent from cross infection among other patients, as infection transmission may occur due to direct contact with infected patients’ secretions.^11^

Although, HSV infection is not straightforwardly associated with mortality but it impairs wound healing and may lead to sepsis.^4^ Several other studies have revealed that EH can be widespread and advance to multiple organ failure and critical life-threatening stage.^6^ In this study, two (2.1%) patients diagnosed with EH developed disseminated EH and died. Derek Y Hsu et al^2^ have mentioned the mortality rate of 0.1% and it is up to 2.7% in severely burned children with Herpes virus infection**3** whereas a study done by Muhammad Sohail and colleagues^6^ in adult population shows mortality rate of 11%. Liaw FY et al^1^ reported the mortality rate as high as 6% to 10% or even 50% in immune compromised adult population.

### Limitations

This is a retrospective study of a single center, and focus mostly on patients residing in Lahore. The other limitation is short follow up of one month only.

## CONCLUSION

EH occurred in 2.4% of admitted pediatric burn patients. Intravenous acyclovir was effective in 97.9% of the patients, although 2.1% developed disseminated EH and expired. Re-activation occurred in 6.4% of the cases and was associated with prolonged hospital stay.

### Authors Contribution:

**FN:** Conceived, prepared manuscript, final approval and is accountable for integrity of the study.

**SR:** Collected data, wrote synopsis, and approved manuscript.

**ARR:** Designed, did statistical analysis, and approved manuscript.

**HMA:** Reviewed, final approval of manuscript.
